# Ultra-Processed Foods and Mental Health in Children and Adolescents: Evidence from a Systematic Review

**DOI:** 10.3390/nu18060899

**Published:** 2026-03-12

**Authors:** Antonia Georgiou, Stavri Chrysostomou, Maria Kantilafti

**Affiliations:** Department of Life Sciences, School of Sciences, European University Cyprus, Nicosia 2404, Cyprus; ag232580@students.euc.ac.cy (A.G.); s.chrysostomou@euc.ac.cy (S.C.)

**Keywords:** ultra-processed foods, UPFs, NOVA classification, mental health, depression, children, adolescents

## Abstract

**Background/Objectives**: The increasing consumption of ultra-processed foods (UPFs) has been associated in recent years with negative effects on both physical and mental health. Ultra-processed products have been increasingly linked with poorer mental health outcomes, with research suggesting associations with higher rates of depression, anxiety, and cognitive difficulties. The aim of this systematic review was to determine whether and to what extent UPF intake is linked to metal health in children and adolescents. **Methods**: The methodological approach involved a systematic review of 20 recent epidemiological studies, identified through the PubMed and EBSCO databases using MeSH and TIAB search terms. The selected articles were evaluated in terms of sample characteristics, assessment tools, results and methodological quality. **Results**: Most findings revealed a positive association between high UPF consumption and mental health problems such as anxiety, depression, irritability or nervousness, sleep disturbances and suicidal ideation. However, variations were observed depending on the country, sex and the assessment tools used. **Conclusions**: In conclusion, the results of this review support the hypothesis that increased consumption of UPFs may be a risk factor for mental health in children and adolescents. Further longitudinal and interventional research is needed, alongside the promotion of healthy dietary policies targeting the pediatric and adolescent populations.

## 1. Introduction

Ultra-processed foods (UPFs) are defined as industrial formulations composed mostly or entirely of substances derived from foods and additives, resulting from a series of industrial processes and typically containing little or no intact whole foods. These products often include ingredients of exclusive industrial use, such as flavorings, emulsifiers and other additives, and are designed to be convenient, highly palatable and long-lasting [1]. According to the NOVA food classification system, foods are categorized into four distinct groups based on the extent and purpose of industrial processing. Group 1 includes unprocessed or minimally processed foods, such as fruits and vegetables, which undergo little or no processing aimed primarily at preservation or minor modifications of texture without substantially altering their nutritional profile [2,3]. Group 2 consists of culinary ingredients extracted from whole foods or directly from nature and used mainly in food preparation and seasoning rather than consumed alone, such as sugar and salt [2,3]. Group 3 includes processed foods produced by combining foods from Group 1 with ingredients from Group 2, including examples such as canned vegetables and salted nuts [3]. Group 4 comprises UPFs, which are industrial formulations made from multiple ingredients and additives designed to enhance flavor, texture and shelf life, such as biscuits and sugar-sweetened beverages [3].

Over recent decades, UPFs have become increasingly prevalent not only in adult diets but also among children and adolescents, while the consumption of unprocessed or minimally processed foods has declined [4]. Evidence from population-based studies indicates that UPFs contribute a substantial proportion of daily energy intake from an early age [1,5,6]. In the United Kingdom, consumption of UPFs at the age of seven accounts for approximately 59.4% of total energy intake [7]. Similarly, in the United States, UPFs contribute approximately 66.2% and 66.4% of total energy intake among children and adolescents, respectively [8].

High consumption of UPFs has been consistently associated with adverse health outcomes and an increased risk of non-communicable diseases in both adults and children [3,9]. Research suggests that UPFs negatively affect glycemic response [10] alter gut microbiota composition [11], reduce overall dietary nutritional quality [12] and are associated with higher prevalence of obesity [13], hypertension [14], cardiovascular diseases and metabolic syndrome [15]. Importantly, the impact of UPFs extends beyond physical health and may also influence mental health outcomes. The World Health Organization (WHO) defines mental health as a state of well-being in which individuals realize their abilities, can cope with the normal stresses of life, work productively and contribute to their community. Mental health encompasses not only the absence of mental disorders but also emotional regulation, resilience and the ability to maintain healthy interpersonal relationships. Common mental disorders include anxiety disorders (such as generalized anxiety disorder, phobias, panic disorder and social anxiety disorder) and depressive disorders (including major depressive disorder and dysthymia), as well as mood disorders (e.g., dipolar disorder), eating disorders (e.g., anorexia nervosa and bulimia nervosa), psychotic disorders (e.g., schizophrenia) and post-traumatic stress disorder (PTSD) [16].

According to the WHO, approximately one in seven adolescents aged 10–19 years experience a mental disorder, accounting for nearly 15% of the global burden of disease within this age group. Depression, anxiety and behavioral disorders are among the most prevalent mental health conditions in youth. Moreover, suicide represents the third leading cause of death among individuals aged 15–29 years worldwide, highlighting the critical importance of identifying modifiable risk factors during childhood and adolescence [16].

Current evidence from two meta-analyses suggests a significant association between UPF consumption and mental health outcomes. Two recent meta-analyses published in 2022 highlight this relationship across different population groups [17,18]. The meta-analysis by Lane et al. (2022), which included 17 observational studies (15 cross-sectional and 2 prospective studies) comprising a total of 385,541 participants (the mean age of the sample was 35 years old), demonstrated that higher consumption of UPFs was associated with an increased likelihood of depressive and anxiety symptoms [18]. Similarly, the meta-analysis conducted by Malmir et al. (2023), focusing exclusively on children and adolescents, analyzed 17 observational studies and reported significant associations among anxiety, sleep disturbances and reduced psychological well-being [17].

Based on the existing literature, an increasing body of evidence suggests a positive association between the consumption of UPFs and adverse mental health outcomes among children and adolescents at a global level. Research interest in this field has grown substantially in recent years. However, the specific impact of UPF consumption on the mental health of younger populations remains insufficiently explored and highly fragmented. Most available studies are observational in nature, vary considerably in their methodological approaches, and differ in terms of mental health outcomes assessed, age groups examined, and definitions of UPF exposure. To date, only a limited number of meta-analyses have examined this association, with only two meta-analyses published so far, both in 2022 [17,18]. However, the meta-analysis by Lane et al. (2022) [18] included predominantly adult populations, while the meta-analysis by Malmir et al. (2023) [17], although focusing on children and adolescents, was based on studies published up to four years prior to its publication. Given the rapid expansion of the literature, the public health relevance of childhood dietary patterns, and the vulnerability of mental health during early life stages, a comprehensive and up-to-date systematic review is warranted to synthesize current evidence, identify research gaps, and inform future research, policy, and intervention strategies.

Therefore, this study aims to examine the association between UPF consumption and mental health outcomes in children and adolescents through a systematic review of the current scientific evidence.

## 2. Materials and Methods

### 2.1. Search Strategy and Eligibility Criteria

A systematic literature search was conducted using PubMed and EBSCOhost databases between November 2026 and January 2026, in order to identify studies examining the association between UPF consumption and mental health outcomes in children and adolescents. The final search was completed on 31 January 2026. Boolean operators (AND, OR) were used to combine keywords and enhance the precision of the search by reducing non-relevant records. In PubMed, the search strategy was refined using both MeSH (Medical Subject Headings) terms and TIAB (title/abstract) field tags to ensure the retrieval of both indexed and recently published studies. In EBSCOhost, the same search terms and Boolean operators were applied, without the use of MeSH or TIAB field tags.

Study selection and data extraction were conducted according to predefined inclusion and exclusion criteria. Eligible studies examined the association between UPF consumption and mental health outcomes and involved children and adolescents aged 3–19 years. Only studies published in English and employing an observational study design, specifically cross-sectional, cohort, and case–control studies, were included. Studies were excluded if they involved animal models, were published in languages other than English, were case reports, or included adults or infants. A summary of the search strategy is presented in Table 1. The full strategy can be found in Supplementary Material Table S1.

### 2.2. Study Selection and Data Extraction

The titles and abstracts identified through the electronic searches were independently screened by two researchers, and full-text articles of studies deemed potentially eligible were retrieved and assessed according to the predefined inclusion criteria. Conflicting assessments were resolved via discussion until a consensus was achieved. From each included study, data were systematically extracted using a standardized form. The extracted information included: author(s), year of publication and country; study design (cross-sectional, cohort or case–control) and follow-up duration (where applicable); sample size and age range of participants; dietary assessment methods and classification of UPFs according to the NOVA system; type and frequency of UPF exposure; mental health outcomes assessed and corresponding measurement instruments; key statistical estimates (e.g., odds ratios, beta coefficients, and confidence intervals); and the main findings of each study.

### 2.3. Quality Assessment

The methodological quality of the included studies was assessed using the Newcastle–Ottawa Scale (NOS), with adaptations according to study design. For cross-sectional studies, scores of 9–10 stars were classified as very high quality, 7–8 stars as high quality, 5–6 stars as satisfactory quality, and 4 stars or fewer as unsatisfactory quality. For cohort studies, methodological quality was categorized as good, moderate, or poor based on domain-specific star allocation. Similarly, case–control studies were classified as having good or poor methodological quality according to predefined star thresholds across the NOS domains [19].

## 3. Results

### 3.1. Main Findings of the Included Studies

**Figure 1:**
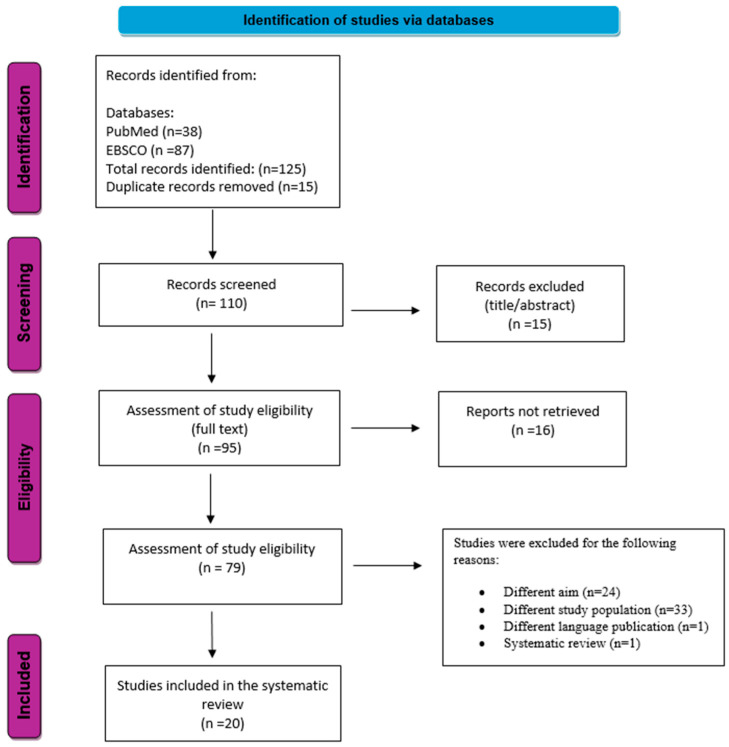
PRISMA flow diagram [20].

The systematic search identified a total of 125 studies (PubMed = 38 and EBSCO = 87). After removing duplicates and screening titles and abstracts, 110 studies proceeded to full-text assessment. Following eligibility assessment, 20 studies met all inclusion criteria and were incorporated into the systematic review (Figure 1).

Most of the studies were cross-sectional designs (*n* = 17), followed by two prospective cohort studies and one case–control study. Sample sizes ranged from 56 to 105,061 participants. The target population comprised children and adolescents aged 3–19 years, with adolescents aged 12–18 years representing the predominant age group. Most studies utilized self-reported questionnaires to assess both dietary intake and mental health status. The primary exposures included consumption of UPFs, junk food and sugar-sweetened beverages. Mental health outcomes encompassed a wide range of indicators, such as depressive and anxiety symptoms, sleep disturbances, suicidal ideation and behavioral problems. More detailed information on the main characteristics of the included studies is presented in Table 2, Table 3 and Table 4.

### 3.2. Methodological Quality

Table 5, Table 6 and Table 7 present the results of the methodological quality assessment of the included cross-sectional, cohort and case–control studies, respectively, using the NOS. Overall, most of the included studies were rated high and very high methodological quality. Most cross-sectional studies demonstrated high [21,22,23,24,25,26,27] and very high methodological quality [28,29,30,31,32,33,34,35,36,37]. The cohort studies were generally assessed as being of good [38] and medium [39] methodological quality. The case–control study [40] was rated as being of moderate methodological quality.

**Table 2:** Study characteristics and main findings of the cross-sectional studies.

Author, Year	Country	Sample	Assessment/Tools	Exposure	Outcome	Main Findings
Vilija et al., 2014 [36]	Lithuania	13–14 years *n* = 1747	Self-administered questionnaire assessing PTSSs, dietary habits and mental health indicators	Frequent consumption of unhealthy foods	PTSSs after traumatic life events	Higher consumption of unhealthy foods was significantly associated with increased PTSSs. Light alcoholic drinks: OR = 1.58, 95% CI: 1.09–2.29; energy drinks: OR = 1.47, 95% CI: 1.07–2.02; soft drinks: OR = 1.44, 95% CI: 1.09–1.92; coffee: OR = 1.49, 95% CI: 1.12–1.98. Consumption of sweets and frozen products was not significantly associated with PTSSs.
Zahra et al., 2014 [35]	United Kingdom	12–16 years *n* = 10,645	Self-reported mental and physical health dietary habits.	Fast food consumption	SDQ	Moderate consumption: OR = 1.31 (95% CI: 1.02–1.65, *p* = 0.03); high consumption: OR = 1.65 (95% CI: 1.27–2.12, *p* < 0.001); daily consumption: OR = 2.00 (95% CI: 1.46–2.75, *p* < 0.001).
Park et al., 2016 [34]	South Korea	12–18 years *n* = 68,043	Dietary behaviors and mental health	Energy drink consumption: ≥5 times/week, <1 time/week and combined consumption of junk food and energy drinks	Sleep disturbance, anxiety, depressive mood, suicidal ideation, suicide planning, and suicide attempts	Frequent energy drink consumption (≥5 times/week) was associated with higher risk of mental health problems: Sleep disturbance: AOR = 1.64 (95% CI: 1.61–1.67); anxiety: AOR = 2.23 (95% CI: 2.19–2.27); depressive mood: AOR = 2.59 (95% CI: 2.54–2.65); suicidal ideation: AOR = 3.14 (95% CI: 3.07–3.21); suicide attempts: AOR = 6.79 (95% CI: 6.59–7.00). Junk food+ energy drinks combination further increased the risk: suicide attempts: AOR = 5.01 (95% CI: 4.89–5.14); suicidal ideation: AOR = 2.55 (95% CI: 2.52–2.59).
Parad et al., 2019 [25]	India	13–16 years *n* = 401	TAI, 24HR, and overall academic performance (school records and MCQ tests)	Fast food consumption	Academic performance and exam anxiety	In the sample from towns, frequent fast food consumption was not significantly associated with increased exam anxiety (r = 0.127, *p* < 0.1), and fast food intake was negatively associated with academic performance (r = −0.125, *p* = 0.012). No significant associations were identified in the rural subgroup (*p* < 0.1).
Jacob et al., 2020 [33]	32 countries: 4 low-income, 13 lower–middle-income, 9 upper-middle-income and 6 high-income	12 years *n* = 105,061	Consumption of fast food, alcohol, sugary drinks, fruits and vegetables, as well as tobacco use and physical activity. Questionnaire for suicides.	Fast food consumption	Suicide attempts in the past 12 months	Higher frequency of fast food consumption was associated with increased odds of suicide attempts. Across countries, 8.3% of adolescents reported suicide attempt. Poorer OR from meta-analysis across 32 countries: OR = 1.31, 95% CI: 1.17–1.46 for higher fast food consumption.
Werneck et al., 2021 [31]	Brazil	14.3 years (mean age) *n* = 99,791	7-day recall GSHS single-item question	Daily consumption of UPFs	Anxiety-related sleep disturbance	Daily UPF consumption was associated with increased likelihood of anxiety-induced sleep disturbance: boys: OR = 1.48, (95% CI: 1.30–1.70); girls: OR = 1.46, (95%CI: 1.34–1.60).
Álvarez-Villaseñor et al., 2020 [26]	Mexico	8–13 years *n* = 406	Questionnaires: EAT for eating anxiety and FFQ for dietary habits and focused on fast food	Fast food consumption	EAT (anxiety triggered by presence of fast food)	No significant association was found between fast food consumption and eating anxiety: OR = 1.61, 95% CI: 0.44–2.3, *p* = 0.95.
Silva et al., 2021 [32]	Brazil	12–17 years *n* = 70,427	GHQ-12	Unhealthy dietary pattern with high UPF consumption and low- or non-processed foods	CMDs	The UPF-rich dietary pattern was associated with higher odds of CMDs: OR = 1.68, 95% CI: 1.51–1.87
Horsager et al., 2022 [23]	Denmark	13–17 years *n* = 423	Online parent-reported questionnaire	UPF-dependence behaviors (food addiction symptoms)	Severity of food addiction symptoms	Higher UPF-dependence scores were observed among adolescents with mental disorders. Mean dependence score: 13.8 (95% CI: 12.6–14.9).
Faisal-Cury et al., 2022 [30]	Brazil	14–15 years *n* = 2680	FFQ and IS-SBQ	UPFs	Internalizing symptoms (anxiety, sadness, and isolation)	Higher UPF consumption was positively associated with internalizing symptoms in both crude (β = 0.14, *p* < 0.001) and adjusted (β = 0.12, *p* < 0.001) models.
Mesas et al., 2022 [29]	Brazil	13–17 years *n* = 94,767	UPF intake, mental health and sociodemographic factors	UPF consumption in the last 24 h	Mental health: depressing feelings, feelings that life is not worth living and that no one cares, and nervousness. Excessive worries about everyday matters.	Higher daily UPF consumption was associated with increased frequency of mental health symptoms: boys: (β = 0.27, 95% CI: 0.03–0.51); girls: (β = 0.31, 95% CI: 0.13–0.50)
Lane et al., 2022 [24]	Iran	12–18 years (girls) *n* = 733	UPF consumption and quality of life	UPF consumption	Quality of life, daytime sleepiness and insomnia	High UPF consumption was associated with increased odds of reduced quality of life (OR = 1.87, 95% CI: 1.13–3.11, *p* < 0.01) and insomnia (OR = 4.04, 95% CI:1.83–8.94, *p* < 0.01). No significant association was found between UPF consumption and daytime sleepiness.
Gketsios et al., 2023 [27]	Greece	10–12 years *n* = 1728	Dietary habits, consumption of UPFs and emotional/behavioral symptoms with emphasis on aggression and feelings of loneliness	Combined consumption of soda drinks and sweet/salty snacks, differentiated by intake level (low vs. at least moderate)	Emotional and behavioral symptoms (which worsen in aggression and loneliness)	Moderate consumption of sweets/salty snacks was associated with higher odds of aggressive behavior (OR = 1.50, 95% CI: 1.19–1.88) and feelings of loneliness (OR = 1.56, 95% CI: 1.20–2.01). Moderate consumption of soda drinks was associated with increased odds of aggression (OR = 1.15, 95% CI: 1.15–1.81) and loneliness (OR = 1.81, 95% CI: 1.41–2.32). The combined model (moderate soda + salty/sweet snacks) showed positive associations with loneliness (OR = 2.36, 95% CI: 1.88–3.25) and aggression (OR = 1.90, 95% CI: 1.53–2.45). In normal-weight children, moderate intake of both soda and salty/sweet snacks was associated with higher odds of aggression (OR = 1.52, 95% CI: 1.31–2.03) and loneliness (OR = 1.92, 95% CI: 1.44–2.35). Obese children had markedly higher odds of aggression (OR = 3.75, 95% CI: 2.38–4.81) and loneliness (OR = 3.70, 95% CI: 2.58–6.61); association was significant (aggression: *p* < 0.002; loneliness: *p* < 0.001).
Gratão et al., 2024 [37]	Brazil	12–17 years *n* = 71,553	Dietary patterns, UPF consumption and mental health symptoms	UPF consumption	Mental health disorders: anxiety symptoms, depressive symptoms and somatic complaints	Higher UPF consumption was positively associated with increased risk of mental health disorders. Adolescents in the highest quartile of UPF consumption had higher odds of mental health problems (OR = 1.20; 95% CI:1.18–1.22).
Rurgo et al., 2024 [21]	Italy	9–18 years *n* = 56 AN	CDI-2 MASC-2 SR SDSC 24HR categorized according to NOVA	UPF consumption	Depressive symptoms, anxiety symptoms and sleep disturbance	Participants with high UPF consumption (≥2 times/day) were at higher risk of depressive symptoms CDI-2 (*p* = 0.011). High UPF intake was also associated with greater nighttime sleep disturbances (nocturnal hyperhidrosis) according to the SDSC (mean = 2.94, SD = 1.48, vs. 2.01, SD = 0.86, *p* = 0.026).
Huang et al., 2025 [22]	China	3–7 years: 18 kindergartens in Wuxi, *N* = 3727	SDQ, DDS and 24HR	UPF consumption	Emotional and behavioral problems: hyperactivity, difficulties in peer relationships and social withdrawal	Daily UPF consumption was associated with increased risk of psychological problems in young children (OR = 1.202, 95% CI: 1.051–1.376).
Yang et al., 2026 [28]	China	10–19 years, *N* = 24,711	Psychological distress: SCL-90 and UPF Intake	UPF consumption	PD	Νο PD: High UPF consumption increased danger of PD (OR = 1.710, 95% CI: 1.486–1.968, *p* < 0.001. High PD: High UPF consumption increased danger (OR = 1.179, 95% CI: 1.054–1.319, *p* < 0.001). High internalized PD: High UPF consumption increased danger (OR = 1.226, 95% CI: 1.050–1.432, *p* < 0.001).

Abbreviations: n: number; CI: confidence interval; PTSS: post-traumatic stress symptom; SDQ: Strengths and Difficulties Questionnaire; OR: odds ratio; AOR: adjusted odds ratio; TAI: Test Anxiety Inventory; 24HR: 24 h Dietary Recall; MCQ: multiple choice question; GSHS: Global School-based Student Health Survey; UPF: ultra-processed food; EAT: Eating Attitudes Test; FFQ: Food Frequency Questionnaire; CMD: common mental disorder; IS-SBQ: Internalizing Symptoms—Social Behaviour Questionnaire; AN: anorexia nervosa; CDI-2: Child Depression Inventory—2nd Edition; MASC-2 SR: Multidimensional Anxiety Scale for Children—2nd Edition, Self-Report; SDSC: Sleep Disturbance Scale for Children; DDS: Dietary Diversity Score; SCL-90: Symptom Checklist-90; PD: psychological distress.

**Table 3:** Study characteristics and main findings of the cohort studies.

Author/Year	Country	Sample	Assessment	Duration	Exposure	Outcome	Main Findings
Davison et al., 2021 [38]	Ireland	13–14 years *n* = 1208	sWEMWBS, KS-10, FAS and FFQ	2 years	Frequency of consumption: junk food, fruits/vegetables, meat, bread/dairy, and protein	Mental well-being and HRQoL	Higher consumption of UPFs/junk food was negatively associated with quality of life (KS-10: Est = −0.165, *p* < 0.001). Although the association with sWEMWBS was not statistically significant in the multivariable model (Est = −0.027, *p* = 0.096), in the structural model, negative association with junk food intake, as well as stability of dietary patterns over time, was observed.
Peacock et al., 2011 [39]	United Kingdom	*n* = 12,942	FFQ and SDQ	16 months	‘Junk food’ consumption	Mental health: behavioral changes	No strong evidence of an association between junk food consumption and behavioral problems. The total difficulties score showed no significant association (OR = 1.05 (95% CI: 0.92–1.21, *p* = 0.45)).

Abbreviations: sWEMWBS: Short Warwick–Edinburgh Mental Well-Being Scale; KS-10: KIDSCREEN-10 Index; FAS: Family Affluence Scale; FFQ: Food Frequency Questionnaire; HRQoL: health-related quality of life, Est: Estimate, SDQ: Strengths and Difficulties Questionnaire.

**Table 4:** Study characteristics and main findings of the case–control study.

Study	Country	Sample	Assessment/Instruments	Exposure	Outcome	Results
Kim et al., 2015 [40]	Korea	12–18 years (girls only), *n* = 849	K-BDI FFQ	Consumption of fast food and UPFs	Presence or absence of depression (score > 16)	Positive association between fast food consumption and depression (OR: 1.88, 95%CI: 1.13–3.14, *p* < 0.001); UPFs food consumption was also associated with higher odds of depression (OR: 2.16, 95% CI: 1.14–3.62, *p* < 0.05).

Abbreviations: K-BDI: Korean version of the Beck Depression Inventory; FFQ: Food Frequency Questionnaire; UPF: ultra-processed food.

**Table 5:** Quality assessment of studies on the association between UPF consumption and mental health in children and adolescents using the Newcastle–Ottawa Scale adapted for cross-sectional studies.

Author/Year	Selection *				Comparability **	Outcome		Total Score
	(1) Representativeness of the Sample	(2) Sample Size	(3) Non Respondents	(4) Ascertainment of Exposure	(1) Control for Confounding Factors	(1) Assessment of Outcome	(2) Statistical Test	
Vilija et al., 2014 [36]	☆	☆	☆	☆	☆☆	☆	☆☆	9/10
Zahra et al., 2014 [35]	☆	☆	-	☆	☆☆	☆	☆☆☆	9/10
Park et al., 2016 [34]	☆	☆	☆	☆	☆☆	☆	☆ ☆	9/10
Parad et al., 2019 [25]	-	☆	☆	☆	☆ ☆	☆	☆ ☆	8/10
Jacob et al., 2020 [33]	☆	☆	☆	☆	☆ ☆	☆	☆ ☆	9/10
Werneck et al., 2021 [31]	☆	☆	-	☆	☆ ☆	☆	☆ ☆	9/10
Álvarez-Villaseñor et al., 2020 [26]	-	☆	☆	☆	☆	☆	☆ ☆	7/10
Silva et al., 2021 [32]	☆	☆	-	☆	☆ ☆	☆	☆ ☆	8/10
Horsager et al., 2022 [23]	-	☆	-	☆	☆ ☆	☆	☆ ☆	7/10
Faisal-Cury et al., 2022 [30]	☆	☆	☆	☆	☆ ☆	☆	☆ ☆	910
Mesas et al., 2022 [29]	☆	☆	☆	☆	☆ ☆	☆	☆ ☆	9/10
Lane et al., 2022 [24]	-	☆	-	☆	☆ ☆	☆	☆ ☆ ☆	8/10
Gketsios et al., 2023 [27]	☆	☆	-	☆	☆ ☆	☆	☆ ☆	8/10
Gratão et al., 2024 [37]	☆	☆	-	☆	☆ ☆	☆	☆ ☆ ☆	9/10
Rurgo et al., 2024 [21]	-	-	-	☆	☆ ☆	☆	☆ ☆ ☆	7/10
Huang et al., 2025 [22]	☆	☆	-	☆	☆ ☆	☆	☆ ☆	8/10
Yang et al., 2026 [28]	☆	☆	☆	☆	☆ ☆	☆	☆ ☆	9/10

Note: Studies with scores of 9–10, 7–8 and 5–6 stars were considered to be of very high, high and satisfactory quality respectively. Four stars or fewer indicate unsatisfactory quality. * One star may be awarded for each numbered item with the Selection and Exposure categories. ** A maximum of two stars may be awarded for Comparability.

**Table 6:** Quality assessment of studies on the association between UPF consumption and mental health in children and adolescents using the Newcastle–Ottawa scale adapted for cohort studies.

Authors/Year	Selection				Comparability	Outcome			Total Score
	(1) Representativeness of the Exposed Cohort	(2) Selection of the Non-Exposed Cohort	(3) Ascertainment of Exposure	(4) Demostration That the Outcome of Interest Was Not Present at the Start of the Study	(1) Comparability of Cohorts on the Basis of the Study Design or Analysis, Controlling for Confounding Factors	(1) Assessment of Outcome	(2) Was Follow-Up Long Enough for Outcomes to Occur	(3) Adequacy of Follow-Up of Cohorts	
Davison et al., 2021 [38]	☆	-	☆	-	☆ ☆	☆	☆	☆	7/9
Peacock et al., 2011 [39]	☆	☆	☆	☆	☆ ☆	☆	☆	☆	9/9

Note: One star may be awarded for each numbered item in the Selection and Outcome/Exposure categories. A maximum of two stars may be awarded for Comparability. Studies with scores of 9–10, 7–8 and 5–6 stars were considered to be of very high, high and satisfactory quality respectively.

**Table 7:** Quality assessment of studies on the association between UPF consumption and mental health in children and adolescents using the Newcastle–Ottawa scale adapted for case–control studies.

Author/Year	Selection				Comparability	Exposure			Total Score
	(1) Adequate Case Definition	(2) Representativeness of the Cases	(3) Selection of Controls	(4) Definition of Controls	(1) Comparability of Cases and Controls on the Basis of the Study Design or Analysis, Controlling for Confounding Factors	(1) Ascertainment of Exposure	(2) Same Method of Ascertainment for Cases and Controls	(3) Non-Response Rate	
Kim et al., 2015 [40]	☆	-	-	-	☆ ☆	☆	☆	☆	6/9

Note: One star may be awarded for each numbered item in the Selection and Exposure categories. A maximum of two stars may be awarded for Comparability. Studies with NOS scores of 0–3, 4–6 and 7–9 were considered to be of low, moderate and high quality respectively.

### 3.3. UPFs and Mental Health—Cross-Sectional Studies

Most of the included cross-sectional studies consistently reported positive associations between UPF consumption and adverse mental health outcomes among children and adolescents. Higher intake of energy drinks, soft drinks, fast food and junk food was associated with increased odds of psychological distress, depressive symptoms, anxiety, suicidal ideation and suicide attempts across diverse populations [27,28,29,30,31,32,33,34,35,36]. These associations were largely consistent across countries, age groups, and specific mental health outcomes.

Several large national studies conducted in Lithuania [36], the United Kingdom [35], South Korea [34], China [28] and Brazil [29,30] demonstrated dose–response relationships, with more frequent consumption linked to progressively higher risks of mental health problems. Particularly strong associations were observed between suicidal behaviors among adolescents and frequent energy drink consumption, especially when combined with junk food intake [34].

Multinational data from the Global School-based Student Health Survey across 32 countries further supported these findings, showing elevated odds of suicide attempts among fast food consumers, with stronger associations in low-income countries [33]. Similar patterns were observed in younger children and specific populations, including preschool-aged children in China [22], adolescent girls in Iran [24], and adolescents with anorexia nervosa in a clinical sample [21].

These findings were consistent across the large majority of cross-sectional studies included in the review, irrespective of country, age group, or the specific mental health outcomes assessed (Table 2).

### 3.4. UPFs and Mental Health—Cohort Studies

Only two cohort studies have examined the longitudinal association between UPFs or junk food consumption and mental health or behavioral outcomes among children and adolescents. The Wellbeing in Schools (WISE) study conducted in Northern Ireland reported a negative longitudinal association between junk food consumption and mental well-being over a two-year follow-up period, with evidence of stability in dietary patterns over time [38]. Higher junk food intake was also associated with poorer overall dietary quality at baseline. In contrast, findings from the Avon Longitudinal Study of Parents and Children (ALSPAC) suggested that although initial analyses indicated positive associations between junk food intake in early childhood and later behavioral difficulties, these associations were attenuated and became non-significant after adjustment for key confounders [39]. Results from the cohort studies are summarized in Table 3.

### 3.5. UPFs and Mental Health—Case–Control Study

Only one case–control study met the inclusion criteria of the present review (Table 4). In this study, higher consumption of UPFs and fast food was positively associated with depressive symptoms among adolescent girls, even after adjustment for potential confounders [40]. Adolescents with greater UPF intake had substantially higher odds of depression, whereas higher consumption of minimally processed plant foods, including vegetables and legumes, was inversely associated with depressive symptoms.

## 4. Discussion

The present systematic review identified reported associations between high UPF consumption and various mental health indicators in children and adolescents across several studies. The most consistent and recurrent associations were observed for anxiety-related symptoms, depressive symptoms and sleep disturbances, which were reported across multiple studies and diverse populations [21,28,29,32,34]. In addition, higher UPF intake was associated with other internalizing and externalizing psychological symptoms, including post-traumatic stress, irritability, nervousness, hyperactivity and emotional detachment [22,30,35,37]. Associations were also reported with suicidal ideation and suicide attempts [33,34], as well as with poorer psychosocial well-being and reduced perceived quality of life [24,38].

In contrast, the cross-sectional study by Álvarez-Villaseñor et al. (2020) [26] and the cohort study by Peacock et al. (2011) [39] found no significant associations. In the study by Álvarez-Villaseñor et al. (2020) [26], no statistically significant association was found between junk food consumption and anxiety. This lack of association is likely due to the exceptionally high prevalence of junk food intake within the sample (94%). Such high prevalence substantially limited exposure and reduced the statistical power of the analysis. Although the assessment of disordered eating-related anxiety using the EAT questionnaire is considered valid and widely applied in adolescent populations, the cross-sectional design precludes causal inference. This limitation arises from the lack of temporal sequencing between exposure and outcome [26]. These findings highlight the need for additional longitudinal research to more accurately evaluate the relationship between junk food consumption and anxiety symptoms in youth. Similarly, the cohort study by Peacock et al. (2011) [39] found no evidence of an association between junk food consumption at 81 months of age and behavioral problems over the following 16 months. Any initial associations were largely attenuated after adjustment for baseline behavioral difficulties and key confounders, including socioeconomic status, child IQ, and maternal mental health. This suggests that the observed relationship may have been influenced by confounding rather than reflecting a direct dietary effect. Age at assessment and methodological factors may also have contributed to the reduced likelihood of detecting diet-related behavioral outcomes [26].

The findings of the present review are consistent with previous systematic reviews and meta-analyses examining the association between ultra-processed food consumption and mental health outcomes. The meta-analysis by Lane et al. (2022), which predominantly included adult populations, reported significant associations between higher UPF intake and increased depressive and anxiety symptoms, findings that align with those observed in the present review [18]. More directly comparable are the results of the meta-analysis by Malmir et al. (2023), which focused on children and adolescents and identified significant associations between junk food consumption, psychological distress and sleep-related outcomes [17]. The present review supports these findings and extends the existing evidence by incorporating more recent studies and examining a broader range of mental health outcomes.

High consumption of UPFs has been associated with poorer mental health outcomes through a rage of biological and psychological mechanisms. UPFs are rich in added sugars, trans fatty acids and other compounds associated with chronic inflammatory processes and gut microbiome dysbiosis, leading to increased intestinal permeability and neuroinflammation [18]. Elevated inflammatory markers such as C-reactive protein (CRP) and cytokines (IL-6, TNF-a) have been repeatedly linked to depressive and anxiety symptoms [41]. Inflammatory processes can disrupt serotonin and dopamine production, affecting mood, sleep and behavior [18]. UPFs may also influence neurotransmission [42]. Chronic consumption may result in deficiencies in essential micronutrients (e.g., magnesium, zinc, and B vitamins) that are required for neurotransmitter synthesis [43]. Excessive sugar intake is associated with dopaminergic dysregulation, characterized by overstimulation of reward pathways, which over time may lead to dependency, emotional instability and mood fluctuations [44]. Evidence from human and animal studies indicates that chronic UPF intake disrupts dopaminergic signaling, increasing cravings and promoting persistent consumption [45]. UPF consumption is also associated with body image disturbances and challenges in weight management, including increased obesity risk and psychological distress related to eating behaviors [45]. Additionally, UPFs are implicated in gut microbiome dysbiosis [46]. Their low fiber content and high levels of processed sugars negatively affect gut microbial composition. Gut dysbiosis may impair gut–brain communication via the vagus nerve, influencing neurotransmitters such as serotonin and dopamine [47]. UPFs also affect psychosocial mechanisms, including emotional eating. Adolescents frequently report using UPFs to cope with stress or loneliness. Social isolation, low self-esteem and the need for immediate gratification increase the preference for ‘comfort foods’, which are often ultra-processed [48].

The current review presents both strengths and limitations. A key strength is that this review specifically focuses on the pediatric population, addressing a critical gap in the literature. To date, only one meta-analysis published in 2022 has examined the association between UPF consumption and mental health in children and adolescents [17]. Additionally, this systematic review incorporates the most recent studies on the topic, providing an up-to-date synthesis of current evidence. By including only studies conducted in children and adolescents, the review allows for a more precise understanding of age-specific associations and developmental considerations that may not be captured in adult-focused research. Unlike earlier meta-analyses, which predominantly focused on adults or relied on older studies, the present review synthesizes a highly fragmented and methodologically diverse body of literature, where mental health outcomes, exposure definitions, and assessment tools vary considerably. By integrating these heterogeneous findings, it offers a coherent and developmentally sensitive perspective on the relationship between UPFs and mental health during critical early life stages. Furthermore, it highlights issues that received limited attention in previous work, such as the overrepresentation of adolescent girls in several studies and the implications for interpretation and generalizability. This age-focused synthesis thus advances the field beyond merely updating available evidence. However, several limitations must be acknowledged. First, the predominance of cross-sectional studies restricts the ability to draw causal conclusions. Only two cohort studies and one case–control study were included, the latter presenting methodological limitations that further constrain causal inference. The heterogeneity of mental health and dietary assessment tools also complicates comparability across studies, despite common use of instruments such as GHQ, SDQ, NOVA classification, FFQs and 24 h recalls. Notably, several of the included studies focused exclusively or predominantly on adolescent girls [21,23,24,40]. This is not incidental, as international data consistently show that adolescent girls report higher rates of anxiety and depression compared with boys after puberty and the WHO highlights that eating disorders and internalized psychological distress are more common in females [49]. However, the overrepresentation of female participants limits the generalizability of the findings to the broader adolescent male population, underscoring the need for balanced gender representation in future studies. Finally, the primary studies did not conduct analyses of macro- or micronutrient profiles, nor did they distinguish between potential ‘toxic’ effects of industrial food processing (e.g., additives and chemical neoformations) and the ‘deficiency’ effects arising from displacement of essential nutrients such as zinc, magnesium, or omega-3 fatty acids. As a result, the mechanisms underlying the observed associations cannot be attributed specifically to nutrient insufficiencies or to processing-related components, and future studies incorporating biochemical markers and detailed nutrient analyses are needed to elucidate these pathways.

## 5. Conclusions

Τhe current evidence indicates a clear association between high consumption of ultra-processed foods and adverse mental health outcomes in children and adolescents. These findings highlight the urgent need for additional prospective studies that can provide stronger causal evidence. Future research should focus on the development and consistent application of age- and culture-specific psychosocial and dietary assessment tools and include diverse socio-cultural populations to enhance validity and generalizability. Beyond research, school- and family-based interventions, along with nutrition education programs, are essential to reducing UPF consumption from an early age and promoting healthier dietary habits. These interventions may contribute to healthier dietary patterns in childhood and potentially support mental well-being. However, further research is needed to clarify causal pathways.

## Figures and Tables

**Table 1:** Keywords and search strategy.

	Keywords	PUBMED	EBSCO
Ultra-Processed Food Exposure	1. Ultra-processed foods	1062	13,543
	2. Highly processed	116	2019
	3. Processed food	2896	45,672
	4. Nova classification	520	2812
	5. Junk food	898	5 777
6. #1 OR #2 OR #3 OR #4 OR #5		5492	69,823
Mental Health Outcome	7. Mental health	277,134	3,768,815
	8. Mental illness	41,524	709,207
	9. Anxiety	308,823	1,638,932
	10. Depression	482,388	2,408,613
	11. Behavioral problems	929,013	128,639
12. #7 OR #8 OR #9 OR #10 OR #11		2,038,882	8,654,206
Children and Adolescents	13. Children	72,718	8 628 693
	14. Child	2,264,370	10,177,613
	15. Adolescents	273,742	6,361,726
	16. Adolescent	2,323,806	6,361,726
17. #13 OR #14 OR #15 OR #16		4,660,894	315,229,758
Study Design	18. Cohort studies	2,738,680	2,057,677
	19. Case–control studies	1,598,669	1,079,214
	20. Cross-sectional studies	541,626	2,281,127
	21. Cohort	951,831	3,648,014
	22. Case–control	172,884	1,355,903
	23. Cross-sectional	626,742	2,787,652
24. #18 OR #19 OR #20 OR #21 OR #22 OR#23		6,630,432	13,209,587
25. #6 OR #12 OR #17 OR #24		13,335,700	337,093,551
		38 records	87 records

Abbreviations: #: Search set number.

## Data Availability

No new data were created or analyzed in this study. Data sharing is not applicable to this article.

## Supplementary Materials

The following supporting information can be downloaded at: https://www.mdpi.com/article/10.3390/nu18060899/s1, Table S1: Full search strategy for each database.
