# Supplementary material for: Ultra-Processed Foods and Mental Health in Children and Adolescents: Evidence from a Systematic Review
Source: Nutrients. 2026 Mar 12;18(6):899. doi: 10.3390/nu18060899 (PMC13029543; doi:10.3390/nu18060899)
Supplement: Supplementary file 1 [file nutrients-18-00899-s001.zip › nutrients-4168732-supplementary.pdf]

Supplementary Table S1: Full search strategy for each database

Database	Last search date	Search fields/syntax	Filters/limits applied	MeSH Terms/Tiab used/keywords used	Full search strategy
PubMed	31 Jan. 2026	MeSH +Title/Abstract (TIAB). Boolean AND/OR	No filters /limits applied (Publications up to 31 Jan 2026)	Keywords: Ultra processed foods [tiab] Highly processed[tiab] Processed food [tiab] Nova classification[tiab] Junk food[tiab] Mental health[tiab] Mental illness[tiab] Anxiety[tiab] Depression[tiab] Behavioral problems[tiab] Children[tiab] child[MeSH Terms] adolescents[tiab] adolescent [MeSH Terms] Cohort studies[MeSH Terms] Case-control studies[MeSH Terms] Cross sectional studies[MeSH Terms] Cohort[tiab] Case-control[tiab] Cross-sectional	(("ultra-processed food"[tiab] OR "ultra processed food"[tiab] OR "highly processed"[tiab] OR "processed food"[tiab] OR "NOVA classification"[tiab] OR "junk food"[tiab]) AND ("mental health"[tiab] OR "mental illness"[tiab] OR "anxiety"[tiab] OR "depression"[tiab] OR "behavioral problems"[tiab]) AND ("children"[tiab] OR "child"[MeSH Terms] OR "adolescents"[tiab] OR "adolescent"[MeSH Terms]) AND ("cohort studies"[MeSH Terms] OR "case-control studies"[MeSH Terms] OR "cross-sectional studies"[MeSH Terms]))
EBSCOhost (All available databases via institutional access)	31 Jan. 2026	Boolean AND/OR. No MeSH/TIAB tags used	Peer reviewed; Journals only (Source type: Academic journals); No limits applied (Publications date up to 31 Jan 2026)	N/A (subject headings not used) Keywords: Ultra processed foods, Highly processed, Processed food, Nova classification, Junk food, Mental health, Mental illness, Anxiety, Depression, Behavioral problems Children, Child, Adolescents, Adolescent, cohort studies,case-control studies,cross sectional studies, cohort, case control, cross-sectional	(ultra processed food OR highly processed OR Processed food OR NOVA classification OR junk food) AND (mental health OR mental illness OR anxiety OR depression OR behavioral problems) AND (children OR child OR adolescents OR adolescent) AND (cohort studies OR case control studies OR cross-sectional studies OR cohort OR Case-control OR Cross-sectional)
